# Elastic Turbulence of Aqueous Polymer Solution in Multi-Stream Micro-Channel Flow

**DOI:** 10.3390/mi10020110

**Published:** 2019-02-07

**Authors:** Jiayan Tai, Yee Cheong Lam

**Affiliations:** School of Mechanical and Aerospace Engineering, Nanyang Technological University, 50 Nanyang Avenue, Singapore 639798, Singapore; JYTai@e.ntu.edu.sg

**Keywords:** elastic turbulence, microfluidics, polymer solution, multi-stream micro-channel flow, contraction-expansion flow

## Abstract

Viscous liquid flow in micro-channels is typically laminar because of the low Reynolds number constraint. However, by introducing elasticity into the fluids, the flow behavior could change drastically to become turbulent; this elasticity can be realized by dissolving small quantities of polymer molecules into an aqueous solvent. Our recent investigation has directly visualized the extension and relaxation of these polymer molecules in an aqueous solution. This elastic-driven phenomenon is known as ‘elastic turbulence’. Hitherto, existing studies on elastic flow instability are mostly limited to single-stream flows, and a comprehensive statistical analysis of a multi-stream elastic turbulent micro-channel flow is needed to provide additional physical understanding. Here, we investigate the flow field characteristics of elastic turbulence in a 3-stream contraction-expansion micro-channel flow. By applying statistical analyses and flow visualization tools, we show that the flow field bares many similarities to that of inertia-driven turbulence. More interestingly, we observed regions with two different types of power-law dependence in the velocity power spectra at high frequencies. This is a typical characteristic of two-dimensional turbulence and has hitherto not been reported for elastic turbulent micro-channel flows.

## 1. Introduction

In fluid dynamics, turbulence is a phenomenon that is associated with highly disordered motion and is conventionally recognized as an inertia-driven phenomenon that occurs at high Reynolds number (*Re* > 2300 in pipe flows) i.e., high-Re turbulence. Its characteristics include good mixing, large velocity fluctuations, and a Kolmogorov’s scaling in the inertia-subrange etc. However, studies have shown that these characteristics can also be manifested when elasticity is introduced into a flow under negligible inertia conditions. The elasticity originates from polymer molecules, which are typically dissolved at minute concentrations (≈0.1–2 wt%) in a solvent, e.g., water; the mixture is termed as an elastic liquid. When an elastic liquid is subjected to high shear and extensional rates in micro-channels, the polymer molecules become stretched and cause a buildup of elastic stresses within the bulk liquid. These elastic stresses can be subsequently released through the relaxation of the stretched polymer molecules. Indeed, our recent investigation has directly visualized and quantified the stretching and relaxation of these polymer molecules [1]. Beyond a critical threshold, the elastic forces (i.e., due to stress accumulation and subsequent relaxation) become sufficiently amplified to dominate over inertia and viscous forces, resulting in the onset of flow instabilities. This phenomenon is termed as ‘elastic turbulence’ and was first introduced in the 1960s to describe polymer melt fracture [2], and later on in the 1970s to illustrate instabilities in Couette flows [3]. Since then, elastic instabilities and turbulence have been reported extensively in experiments of rotating plate flows [4,5,6], Couette–Taylor flows [7,8,9], cross-slot flows [10,11], curvilinear flows [12,13,14,15], and contraction/expansion flows [1,16,17,18,19,20,21,22]. The phenomenon has also been greatly exploited in the last decade to enhance mixing in micro-channels [12,15,18,23,24], which is usually difficult due to the low-Re restriction.

Early studies on elastic turbulence date back to the 1970s and focused primarily on visual observations of flow transitions in contraction flows e.g., Newtonian like flow, diverging flow, periodic flow, non-periodic flow, and unstable flow [16,17,20,25,26,27,28]. Due to the lack of any statistical comparison with that of high-Re turbulence, the aptness of the term ‘turbulence’ in describing the phenomenon was met with considerable debate, as this opposed the conventional belief that a high-Re criteria was necessary. Instead, the term ‘instability’ was more commonly employed and accepted. However, Groisman and Steinberg [4] observed a power law decay (i.e., with a −3 slope) in the velocity power spectra of an unstable elastic rotating flow (*Re* = 0.3). This is a known statistical characteristic of high-Re turbulence, and the term ‘elastic turbulence’ was used to describe the phenomenon observed. Thereafter, statistical approaches have been adopted in the analyses of elastic turbulence, and the power law decay in the velocity spectra was also observed widely [1,15,21,24,29]. However, these investigations were mostly limited to single-stream flows comprising of one liquid type.

Lam’s group [1,18,19,22] was the first to introduce a 3-stream flow, comprising of two dissimilar liquids, into a contraction-expansion micro-channel. They observed the unstable flow of a highly elastic center-stream liquid downstream of the contraction exit, which appeared to be turbulent in two dimensions (2D). This was made possible due to the ‘free space’ (or relatively easily deformable space) created by two low viscosity side-stream liquids. The unstable downstream flow also led to high mixing efficiencies, while upstream mixing was negligible [18]. However, in-depth statistical analyses of the downstream flow field were not conducted.

Here, we investigate the characteristics of the 3-stream flow using the same contraction-expansion configuration, by employing the particle image velocimetry (PIV) technique. Since interesting flow dynamics due to relaxation of the highly elastic center-stream liquid had only been observed downstream, this study will only focus on the flow field downstream beyond the contraction exit. Various statistical techniques and flow field visualization tools are presented, and comparisons of the flow field statistics are compared with that of high-Re turbulence whenever appropriate. Hitherto, such a comprehensive statistical analysis and visualization of an elastic turbulent flow field has yet to be reported for a multiple-stream contraction-expansion micro-channel flow. The interactions between dissimilar liquid streams in these flows have been reported to greatly enhance mixing [18,19,22], and the current study aims to uncover the underlying mechanisms and turbulent flow characteristics, which are rather different as compared to that of a single-stream flow. This investigation could also be useful in micro-channel cooling applications where single stream elastic turbulent flows have recently been reported to significantly enhance heat transfer [30,31,32].

## 2. Materials and Methods

### 2.1. Micro-Channel

**Figure 1:**
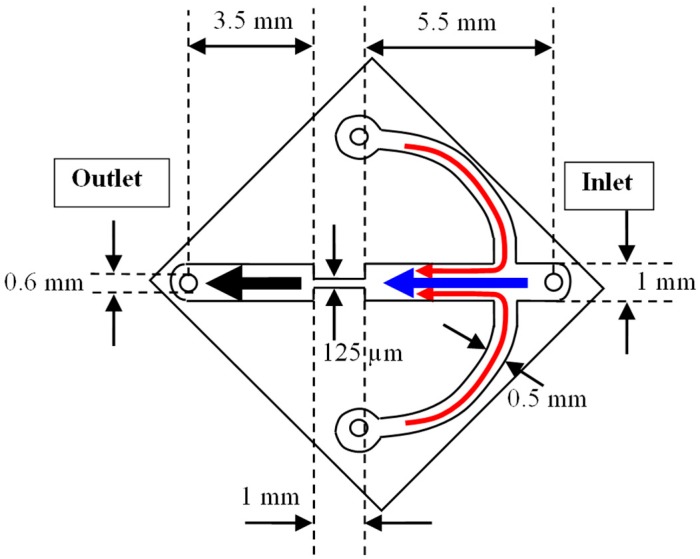
Schematic 2-D diagram of the micro-channel. Arrows depict the flow of the center-stream (blue) and side-stream (red) liquids respectively.

Figure 1 shows a schematic diagram of the micro-channel for our investigations. The micro-channel was fabricated by etching a bottom silicon layer using Deep Reactive Ion Etching (DRIE), and subsequently bonded to a top Pyrex glass layer via anodic bonding. The transparency of the glass allowed ease of image capturing for subsequent PIV analyses of the flow field. The micro-channel consists of 3 inlets (i.e., one center-stream liquid and two side-stream liquids) and 1 outlet (see Figure 1). As the liquids flowed towards the outlet, they passed through a sudden contraction (8:1:8) which is 1 mm in length and has a width of 125 µm. The whole micro-channel has a depth of 180 µm, and the flow field images were captured at mid-channel height i.e., 90 µm. In all experimental runs presented here, the flow from each side-stream liquid contributed to a quarter (i.e., 25%) of the total volume flow rate. More details on the flow setup can be found in our previous publication [18].

### 2.2. Test Liquids

**Figure 2:**
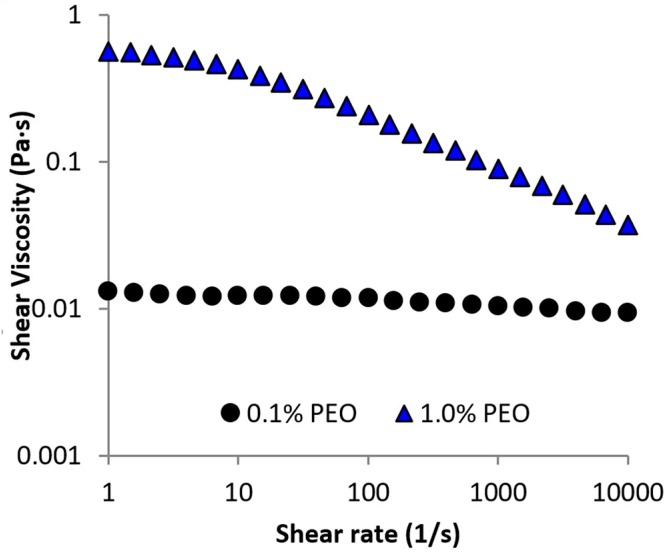
Viscosity plot of test liquids across a wide range of shear rate.

**Figure 3:**
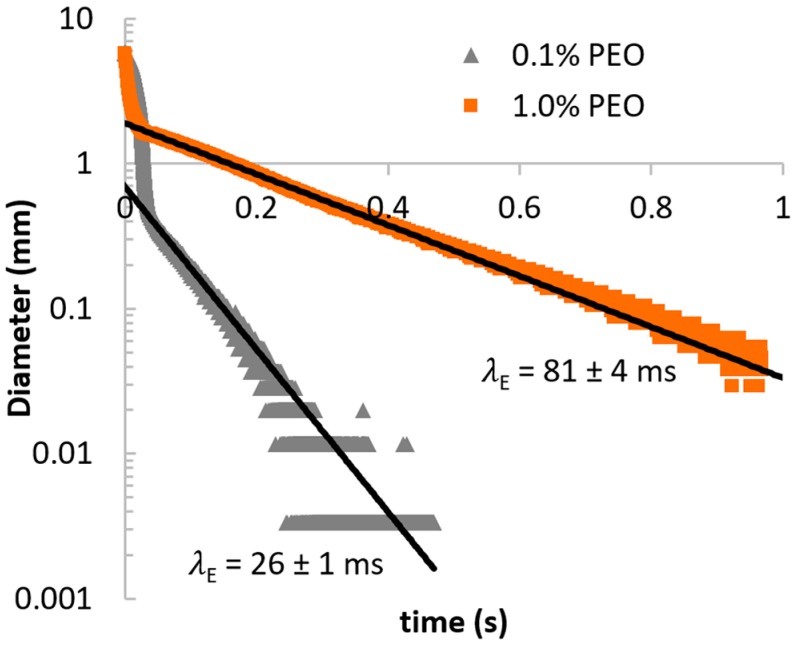
Plot of filament diameter over time as measured by CaBER for the test liquids. The fitted black line was used to obtain the extensional relaxation time for each liquid.

The test liquids were obtained by dissolving polyethylene oxide (PEO) with an average molecular weight of 2 × 10^6^ g/mol (POLYOX, The Dow Chemical Company, Midland, MI, USA) into an aqueous 55 wt% glycerol solvent. Similar to our previous publications [18,19,22], the center-stream liquid consists of 1 wt% PEO and is thus relatively highly elastic, while the side-stream liquid consists of 0.1 wt% PEO and has a much lower elasticity as compared to the center-stream liquid due to the reduced polymer concentration. As the size of the elastic polymer molecules were too minute for proper flow visualization and characterization, seeding particles (3 µm, Thermo Scientific, Waltham, MA, USA, 0.1 wt%) were added into both test liquids to facilitate PIV tracking for proper flow visualization. Two precision pumps (KD Scientific, Holliston, MA, USA) were used to infuse the test liquids, one for the center-stream liquid (KDS 410) and one for the two side-stream liquids (Legato^®^ 210) respectively. Table 1 shows the composition of the test liquids. The shear viscosity (*η*) and extensional relaxation times (*λ_E_*) of the liquids were measured using the Gemini Hr Nano Rheometer and the Capillary Breakup Extensional Rheometer (CaBER) respectively and are shown in Figure 2 and Figure 3 respectively. *λ_E_* was measured to be ≈81 ms for the center-stream liquid, and ≈26 ms for the side-stream liquid. In general, the relaxation time of the individual polymer molecules is shorter than *λ_E_*, since CaBER measures the longest relaxation time in the liquid.

### 2.3. Optical Setup

**Figure 4:**
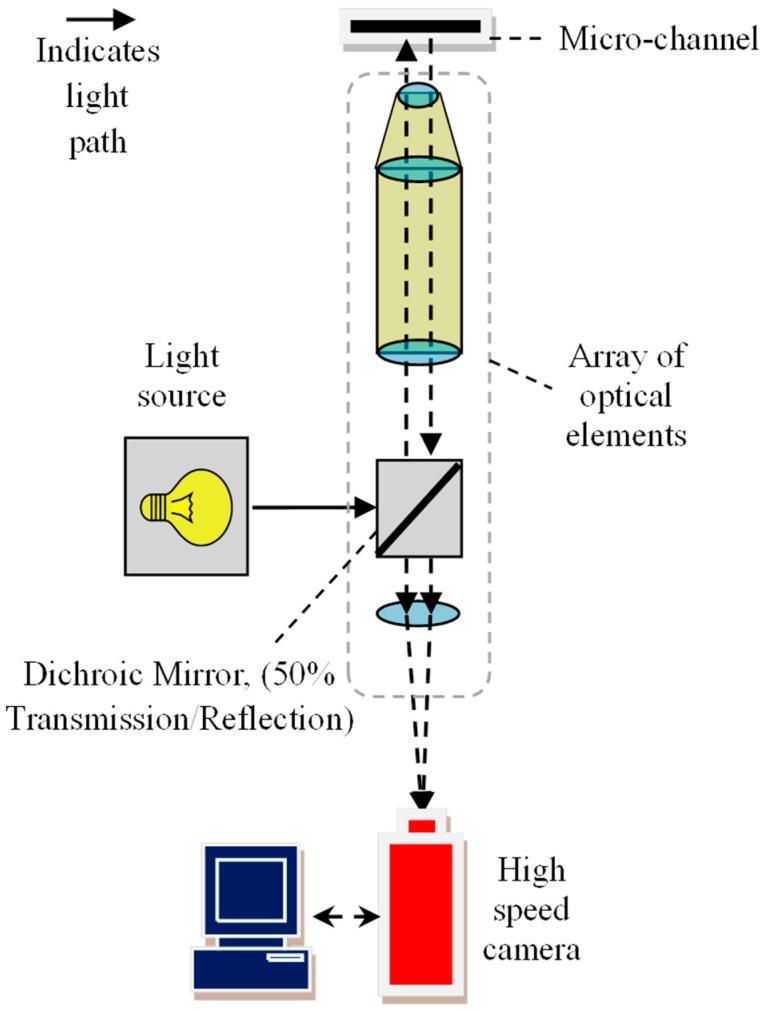
Schematic diagram of optical setup.

**Figure 5:**
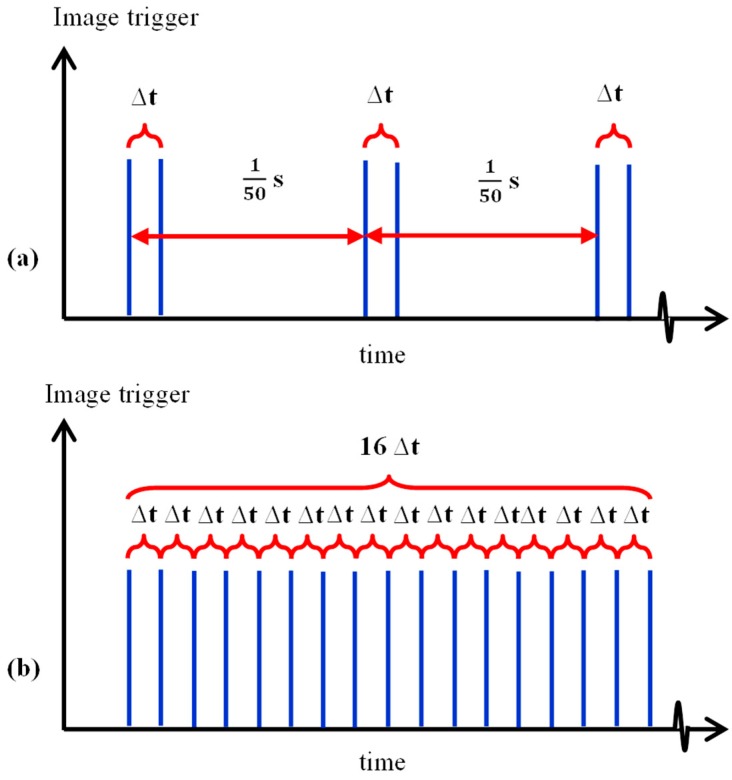
Schematic diagram of the two recording modes. (**a**) Mode 1. (**b**) Mode 2.

Figure 4 shows a schematic diagram of the optical setup. The micro-channel was observed through a 10× telescopic lens (Optem Zoom 70 XL, depth of field = 22 µm, Qioptiq, Rhyl, UK), and images were captured with a high speed camera (Photron SA5). The experiments were conducted at two flow rates i.e., 2 mL/h and 20 mL/h. For each flow rate, images of the downstream flow field were captured using two recording modes i.e., recording mode 1 (low frequency) and mode 2 (high frequency). In mode 1 (see Figure 5a), the camera was triggered by a function generator at 50 Hz bursts, over 200 s. When triggered, the SA5 was programmed to capture 1 image pair i.e., 2 images separated by a time interval (Δ*t*). Mode 1 enabled images to be captured over a longer period of time, such that the average velocity profiles of the flow field could be evaluated using PIV. In contrast, mode 2 was used to continuously record images at high frequencies (see Figure 5b), and images were captured at time intervals (Δ*t*) similar to that in mode 1. Mode 2 was used to measure high frequency velocity changes, i.e., to detect events with short time scales, which should be on the order of the molecular relaxation time of the center-stream liquid.

To obtain a measure of the inertia and elastic forces in the flow field, the Reynolds number (Re) and Deborah number (De) have been quantified. Here, we adopt the definitions of Re and De similar to our previous publications [18,19,22]. The Reynolds number is defined as *Re* = 2*ρQ*/*η*(*w_c_* + *d*), where *ρ* is the liquid density, *Q* is the flow rate, *η* is the solution shear viscosity in the contraction, *w_c_* is the average contraction width occupied by each liquid stream, and *d* is the micro-channel depth respectively. Since the investigation was focused on the extensional effects in the contraction-expansion flow, the Deborah number is defined as *De* = 2*λ_E_Q*/*w_c_d*^2^, where *λ_E_* is the extensional relaxation time of each liquid. Table 2 shows the two flow rates investigated, the time interval (Δ*t*) used in the image acquisition, and the corresponding average velocity ($\bar{U}$), shear rate ($\dot{\gamma }$), viscosity (*η*), *Re*, *De*, and *El* of each liquid stream in the contraction respectively. The computational methods used to obtain $\bar{U}$, $\dot{\gamma }$, and *η* are explained in the Supplementary Materials (Section S1). From Table 2, it can be observed that *Re* is slightly higher in the lower viscosity side-stream liquids, as compared to the center-stream liquids. Nevertheless, inertial forces were still considerably low (maximum *Re* ~ 1) in all experimental runs.

## 3. Results and Discussion

### 3.1. Mean Flow Statistics

**Figure 6:**
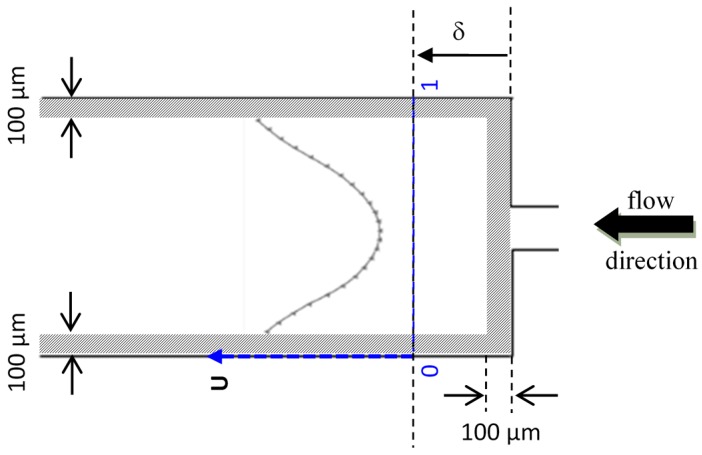
Schematic diagram of the contraction-expansion micro-channel, showing the plot of a mean axial velocity profile at a distance *δ* downstream of the contraction. Data in the shaded region has been removed due to high noise level.

Figure 6 shows a schematic diagram of the downstream region of the micro-channel, where *δ* represents the distance away from the contraction. To compute the flow statistics (i.e., mean velocity, standard deviation etc.), 10,000 image pairs (1.4 mm × 1 mm, 700 × 500 pixels) were captured using recording mode 1, with a time interval (Δ*t*) corresponding to each flow rate as shown in Table 2. Each image pair was subsequently processed using a cross-correlation PIV algorithm [33], with interrogation window sizes of 64 × 32 pixels and a 50% overlap in both axes. For conciseness, the flow statistics at two cross-sections will be presented i.e., at *δ* = 0.3 mm and *δ* = 1.0 mm. Due to the significantly higher velocity and poor illumination conditions near the channel walls, data obtained from within 100 µm of all the channel walls have been omitted.

**Figure 7:**
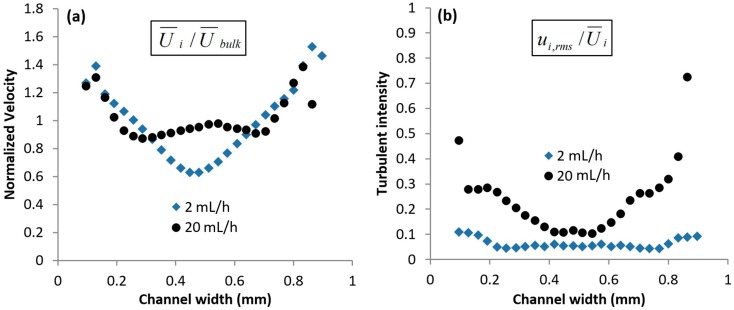
Mean flow statistics at *δ* = 0.3 mm. (**a**) Normalized axial velocity and (**b**) Turbulent intensities.

Figure 7a shows the normalized mean axial velocity profiles for the flow rates at *δ* = 0.3 mm. Each profile was obtained by normalizing the velocity at each interrogation window (${\bar{U}}_{i}$) with the mean velocity at the particular flow rate (${\bar{U}}_{bulk}$). Here, ${\bar{U}}_{bulk}$ is defined as ${\bar{U}}_{bulk}=Q/wd$, where *w* refers to the channel width (i.e., 1 mm). At *Q* = 2 mL/h, the profile is inverted parabolic in nature. The center-stream liquid being highly elastic, exhibits die swell behavior [34] upon exiting the contraction; the die swell effect is a classic and well-known characteristic of viscoelastic liquids [34], whereby a viscoelastic liquid ‘swells’ upon exiting a contraction, after being subjected to high extensional rates. This is due to stress recovery of the polymer molecules in the viscoelastic liquid. Thus, it pushes the side-stream liquids towards the channel walls to occupy a larger space and moves at a lower speed. Due to mass conservation, the side-stream liquids have to flow at higher velocities, resulting in the inverted profile observed. As the flow rate was increased to *Q* = 20 mL/h, a distinct peak could be observed in the center of the velocity profile. Note that *Re* ≈ 0.5, and hence this is not an inertial effect. Instead, the observed peak is due to incomplete relaxation of the extensional stresses that have been stored in the polymer molecules, which had been highly stretched as they flowed past the contraction. This correlation between the extended state of the polymer molecules (i.e., incomplete relaxation) and peak in velocity profile has already been experimentally observed and reported previously [1].

Figure 7b shows the normalized turbulent intensities for both flow rates at *δ* = 0.3 mm. The data was computed by normalizing the standard deviation of the velocity measurements at each interrogation window $\left(u_{i,rms}\right)$ with the mean velocity (${\bar{U}}_{i}$). At *Q* = 2 mL/h, the turbulent intensities in the flow were low, with a slight increase to ≈0.1 near the sidewalls. This is due to a small degree of relaxation of the center-stream liquid, resulting in small fluctuations at the interface between the center-stream and side-stream liquids. As the flow rate was increased to *Q* = 20 mL/h, the turbulent intensities increased significantly. At this flow rate, the polymer molecules had been stretched significantly in the contraction. This caused a large amount of elastic energy to be stored in the center-stream liquid, which was subsequently released via a random ‘sweeping’ motion that was observed downstream of the contraction (see Supplementary Materials for video clip on ‘sweeping’ motion). Thus, the fluctuation at the liquid interfaces increased drastically, resulting in high turbulent intensities (see Figure 7b). Note that such high turbulent intensities are typically only reported for high-Re large channel flows [35,36]. Since the relaxation of stresses in the center-stream liquid at *Q* = 20 mL/h was incomplete (based on deduction from the peak in velocity profile and our previous experimental work [1]), the turbulent intensities at the center were not significantly higher than that at *Q* = 2 mL/h.

**Figure 8:**
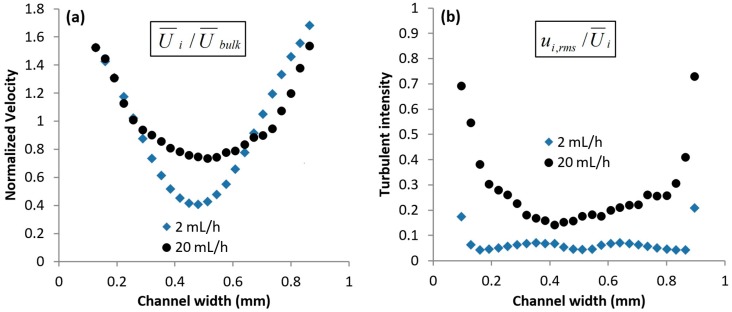
Mean flow statistics at *δ* = 1.0 mm. (**a**) Normalized axial velocity and (**b**) Turbulent intensities.

Further downstream at *δ* = 1.0 mm, the normalized axial velocity profiles retain the same inverted parabolic shape (see Figure 8a). The center peak at *Q* = 20 mL/h had disappeared, indicating that the extensional stresses of the polymer molecules in the center-stream liquid had been released; such a correlation between the velocity profile and the direct observation of the relaxation of polymer molecules had been made previously [1]. The relative differences between the velocity at the center and the side of the micro-channel have also increased at *δ* = 1.0 mm, as compared to that at *δ* = 0.3 mm for both flow rates. This augmented die swell effect is caused by the further relaxation of elastic stresses in the center-stream liquid, which results in a decrease in velocity at the centerline.

The normalized turbulent intensities at *δ* = 1.0 mm (see Figure 8b) appear to be quantitatively similar to that at *δ* = 0.3 mm for *Q* = 2 mL/h. This means that the fluctuations due to the elastic stress relief mechanism of the center-stream liquid have not been reduced by viscous dissipation. At *Q* = 20 mL/h, the turbulent intensities had increased, indicating a delayed relaxation of stresses in the polymer molecules in the center-stream liquid. Thus, this led to an increase in localized fluctuations, which resulted in an overall increase in turbulent intensities as compared to that at *δ* = 0.3 mm. Such high turbulent intensities were absent when Newtonian liquids were employed instead, or when the flow consisted of only one PEO stream (see Section S3 in the Supplementary Materials).

### 3.2. Normalized Reynolds Stresses

**Figure 9:**
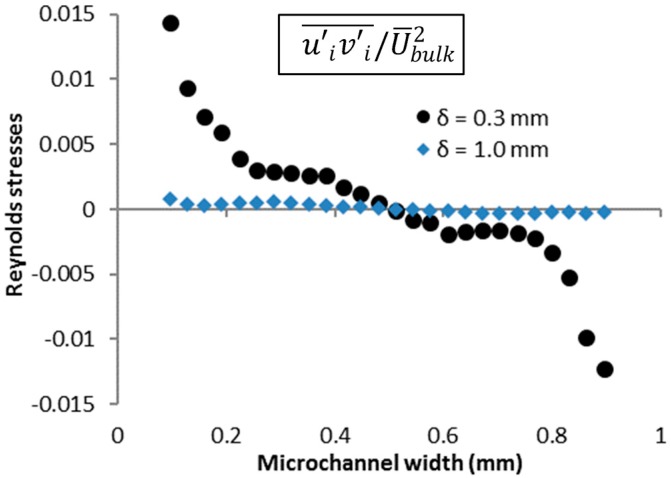
Normalized Reynolds stresses at 20 mL/h across the channel at *δ* = 0.3 mm and *δ* = 1.0 mm.

To study the momentum exchange between the mean flow and the turbulence, the normalized Reynolds stresses were computed for *Q* = 20 mL/h, at *δ* = 0.3 mm and 1.0 mm. Figure 9 shows the normalized Reynolds stresses, computed by normalizing the Reynolds stresses ($\bar{u_{i}^{\prime }v_{i}^{\prime }}$) with the mean axial velocity squared (${\bar{U}}_{bulk}^{2}$). The Reynolds stresses (i.e., momentum flux) can be observed to be highest close to the contraction, i.e., at *δ* = 0.3 mm, due to the random release of stored elastic energy from the highly stretched polymer molecules into the flow. Since the turbulence originates from the center-stream liquid, the momentum flux is zero at the center, and is directed towards the channel walls by the ‘sweeping’ motion. Such high Reynolds stresses are typically observed only in high-Re flows [35,36]. At *δ* = 1.0 mm, the Reynolds stresses are greatly diminished.

### 3.3. Single-Point Flow Statistics

**Figure 10:**
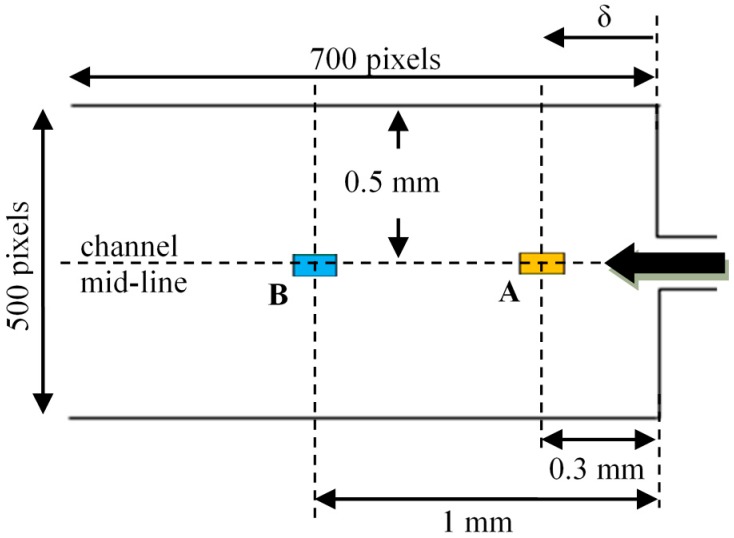
Locations of interrogation windows A and B for single-point velocity measurements.

**Figure 11:**
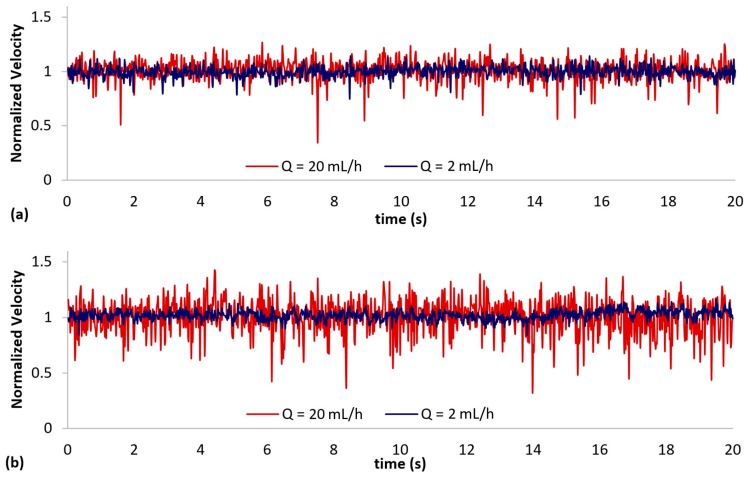
Normalized low frequency axial velocity measurements at (**a**) interrogation window A (*δ* = 0.3 mm) and (**b**) interrogation window B (*δ* = 1.0 mm) obtained using recording mode 1.

To investigate the temporal changes in velocity, single-point axial velocity statistics are presented here. Due to the large amount of data, only PIV measurements at 2 locations along the centerline have been included i.e., *δ* = 0.3 mm, and 1.0 mm. Figure 10 shows the location of the two interrogation windows A and B (64 × 32 pixels, 128 µm × 64 µm), where measurements were obtained at the channel mid-plane i.e., 90 µm height. Figure 11a,b shows the normalized temporal velocity statistics at the 2 interrogation windows. The velocimetry data was first obtained by processing 10,000 image pairs (recording mode 1), and each image pair was separated by a duration of Δ*t* (refer to Table 2). Subsequently, the data at each flow rate was normalized by its mean value for ease of comparison. For conciseness, the plot has been limited to a 20-second period. It can be observed that at *Q* = 2 mL/h, some small-scale and low amplitude fluctuations at both locations are present (see Figure 11a,b). However, as the flow rate was increased to *Q* = 20 mL/h, the fluctuation amplitudes increased, and ‘spikes’ could be observed randomly in time.

**Figure 12:**
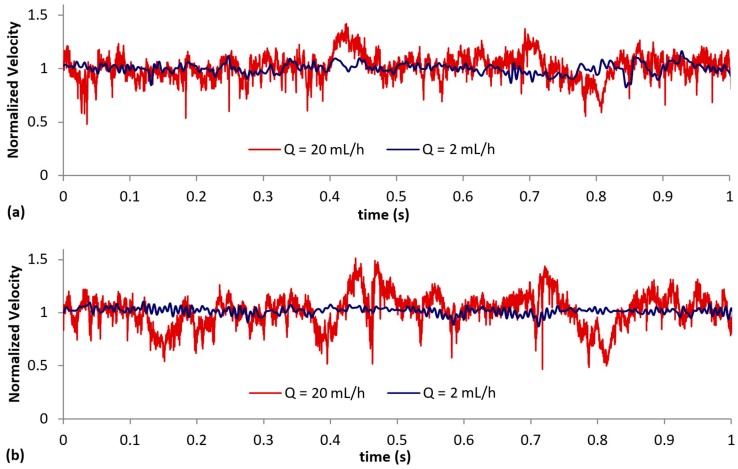
Normalized high frequency axial velocity measurements at (**a**) interrogation window A (*δ* = 0.3 mm) and (**b**) interrogation window B (*δ* = 1.0 mm) obtained using recording mode 2.

Figure 12a,b show the normalized high frequency data measured at both interrogation windows over a 1-second period (recording mode 2). A set of 50,000 images was continuously captured at high frequencies (i.e., 250 Hz and 4000 Hz) for each flow rate (i.e., *Q* = 2 mL/h and 20 mL/h), using the same time interval (Δ*t*) as that in mode 1. Similar to the lower frequency recordings, i.e., mode 1, the flow at *Q* = 2 mL/h is comprised of small scale fluctuations. However, the changes in velocity at *Q* = 20 mL/h are now clearly discernable, as compared to the sudden ‘spikes’ observed in Figure 11a,b due to the lower recording frequency.

### 3.4. Velocity Power Spectra

**Figure 13:**
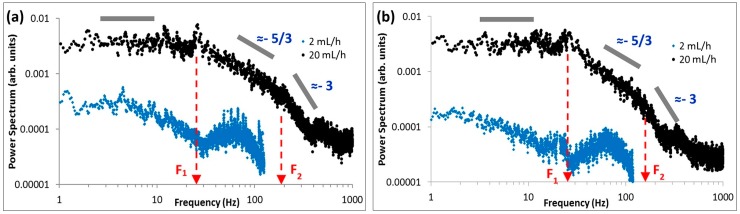
Power spectrum of the axial velocity fluctuations for the two flow rates investigated at (**a**) interrogation window A (*δ* = 0.3 mm) and (**b**) interrogation window B (*δ* = 1.0 mm).

To investigate the distribution of turbulent kinetic energy across various frequencies, the power spectra of the axial velocity fluctuations at interrogation windows A and B (*δ* = 0.3 mm and 1.0 mm) are shown in Figure 13a,b. Due to the Nyquist criteria, the maximum frequency for each flow rate (i.e., 2 mL/h and 20 mL/h) was half the image capturing frequency (i.e., 125 Hz and 2000 Hz respectively). Here, only frequencies above 1 Hz are included because the recording duration was short. At *Q* = 2 mL/h, the fluctuations are small at both interrogation windows, and the velocity power spectrum exhibits a decrease of less than one decade across the whole frequency range. However, as the flow rate increased to *Q* = 20 mL/h, three important features can be observed in the power spectra. The spectra at both interrogation windows are characterized by an almost horizontal plateau at low frequencies, followed by a drop in power across two decades which are characterized by two power law decays with different slopes i.e., −5/3 and −3 (see Figure 13a,b).

The horizontal plateau is observed to range from 1 Hz to ≈20–30 Hz before it decreases at F_1_ (see Figure 13a,b). This drop-off frequency (F_1_) is much higher than that reported by Groisman and Steinberg [4] (≈0.1 Hz) and Bonn et al. [21] (<1 Hz) and is on the order of the lowest frequency at which the center-stream fluid relaxes (i.e., 1/*λ_E_* ≈ 12 Hz). Subsequently, the spectrum follows a power law decay with a slope of ≈−5/3. This is usually observed in the inertia sub-range of high-Re turbulence, whereby energy is transferred from large to small eddies. However, throughout this experiment, inertia forces were relatively low (maximum *Re* ~ 1). A second drop-off frequency (F_2_) can be observed clearly (i.e., at ≈100–200 Hz), whereby the spectrum decays with a second, steeper power law slope of ≈−3. The second slope appears more prominent closer to the contraction exit (i.e., *δ* = 0.3 mm, see Figure 13a), and is slightly attenuated further downstream (i.e., *δ* = 1.0 mm, see Figure 13b). It is interesting to observe a double cascade here. This had also been reported earlier for elastic turbulence in a Couette-Taylor (CT) flow system [7], where the inflection frequencies were found to coincide closely to the driving frequencies of the CT flow i.e., rotation rates. In contrast, the inflection frequencies observed in our findings are nowhere near the driving frequencies of our infusion system. Instead, the presence of a double cascade could indicate the occurrence of 2D turbulence, where F_2_ corresponds to the energy injection point into the turbulence [37,38,39]. Note that here we have invoked the Taylor’s ‘frozen eddy’ hypothesis, which was assumed to be valid (refer to Supplementary Materials). The significance of F_2_ here is yet to be fully understood; however, it is clear that the turbulence observed is driven by elasticity (i.e., molecular relaxation) at frequencies > 12 Hz (i.e., 1/*λ_E_*), since *λ_E_* is a measure of the longest molecular extensional relaxation time in the center-stream liquid.

### 3.5. Flow Field Visualization

**Figure 14:**
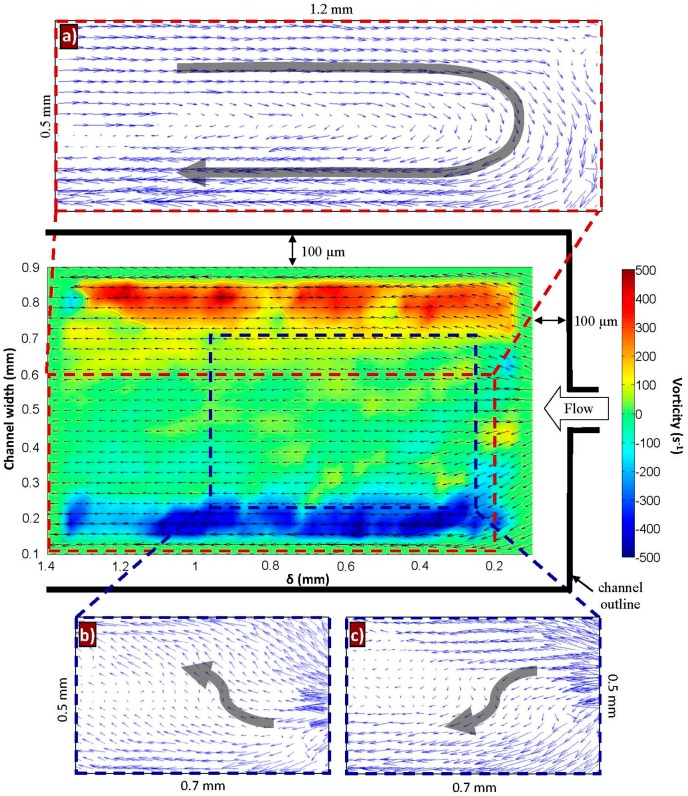
An instantaneous plot of velocity and vorticity (color plot) downstream of the contraction, from *δ* = 0.1–1.4 mm. Insets a–c show structures (drawn to scale) at various time instances located in different regions, obtained using the Galilean decomposition method with a fraction of the convective velocity removed. (**a**) U − ${\bar{U}}_{bulk}$, (**b**,**c**) U − 0.6${\bar{U}}_{bulk}$ (at two different time instants).

Figure 14 shows an instantaneous snapshot of the flow field, downstream of the contraction exit (*δ* = 0.1–1.4 mm), at *Q* = 20 mL/h. The velocity vectors and vorticity color plots (anti-clockwise being positive) have also been included in the figure. Here, the out-of-plane component (i.e., z-component) of vorticity is considered, as determined from the two-dimensional (i.e., x- and y-components) PIV data. The vorticity was computed based on the least squares approach [40], and the flow direction is from right to left.

Due to the faster flowing side-stream liquid, regions of higher vorticity are located closer to the channel walls. To visualize the presence of interesting flow phenomena caused by the elastic stress relaxation of the center-stream liquid, the Galilean decomposition technique was applied [41]. This technique involves subtracting (a fraction of) the mean axial velocity (${\bar{U}}_{bulk}$) from the instantaneous flow field, to unveil non-stationary ‘structures’ or interesting behavior that could have been convected away. Insets Figure 14a–c show the subtracted flow field, where ‘structures’ were observed at various time instants, in different regions of the micro-channel. Note that the figures are not drawn to scale. It is interesting to observe a large eddy (≈1.3 mm × 0.6 mm, see Inset Figure 14a) when ${\bar{U}}_{bulk}$ was completely subtracted. In addition, an upwards (Inset Figure 14b) and downwards (Inset Figure 14c) ‘sweeping’ flow was observed when 0.6 ${\bar{U}}_{bulk}$ was subtracted. Recall that this ‘sweeping’ motion was only possible due to the presence of two low viscosity side-stream liquids, which allowed the elastic center-stream liquid to relax ‘freely’ in the lateral direction.

## 4. Conclusions

By dissolving small quantities of PEO polymer molecules into water, elasticity is introduced into the aqueous solution due to the elastic property of the PEO molecules. This viscous-elastic polymeric solution can behave drastically differently from the viscous aqueous solution. Based on a 3-stream expansion-contraction micro-channel flow configuration, we analyzed the characteristics of its elastic turbulent flow field using PIV. The turbulence was driven by the stretching and relaxation of the highly elastic liquid (namely the polymer molecules), which was subjected to large extensional forces in the contraction, before being allowed to relax freely in the midst of two low viscosity side-stream liquids. At *Q* = 20 mL/h, the flow field exhibited high velocity fluctuations and turbulent intensities, accompanied by large normalized Reynolds stresses with magnitudes comparable to that reported in high-Re turbulence. In addition, the velocity power spectra (at *Q* = 20 mL/h) revealed two regions with two different types of power-law dependence (i.e., −5/3 and −3 power law decay), characterized by two drop-off frequencies (F_1_ and F_2_). This characteristic is similar to that of 2D turbulence, where F_2_ is the energy injection point into the turbulence. The turbulence observed here could be predominantly 2D in nature, due to the 3-stream flow configuration presented which promotes the manifestation of the center-stream liquid in two dimensions. This is also supported by evidence from the Galilean decomposition technique, where an up-down ‘sweeping’ flow was observed. Since elastic turbulence is driven by molecular stretching and relaxation, it would be interesting to investigate in the future, on the possibility of any correlation between F_2_ (i.e., possibly the energy injection point into the turbulence) and *λ_E_* (extensional relaxation time). Furthermore, the contraction length has been reported to affect the upstream flow dynamics in a contraction-expansion flow [20]. This could be further explored, and a correlation study between the downstream and upstream flow dynamics could also be examined.

## Figures and Tables

**Table 1:** Composition of test liquids.

Test Liquid	PEO (g)	Glycerol (g)	Water (g)	Microsphere (g)
Center-stream	1.00	55	44	0.03
Side-stream	0.10	55	44	0.03

**Table 2:** Experimental flow parameters and corresponding liquid properties in the contraction.

	Properties	Extensional Relaxation Time (ms)	Flow Rate (mL/h)	Δ*t* (s)	Mean Velocity ($\bar{U}$, m/s)	Shear Rate ($\dot{\gamma }$, s^−1^)	Viscosity (η, Pa·s)	*Re*	*De*
Fluid									
Center-stream Liquid		81 ± 4	2	0.004	0.03	364	0.126	0.022	27
			20	0.00025	0.31	4262	0.052	0.528	278
Side-stream Liquid		26 ± 1	2	0.004	0.02	1107	0.01	0.145	6
			20	0.00025	0.21	10514	0.01	1.45	59

## Supplementary Materials

The following are available online at http://www.mdpi.com/2072-666X/10/2/110/s1, text file: Supplementary_Materials.pdf and video clip: Sweeping_Motion.mp4 which support the concepts discussed in this paper.

## References

[1] Tai J., Lim C.P., Lam Y.C. (2016). Visualization of polymer relaxation in viscoelastic turbulent micro-channel flow. Sci. Rep..

[2] Vinogradov G.V., Ivanova L.I. (1968). Wall slippage and elastic turbulence of polymers in the rubbery state. Rheol. Acta.

[3] Giesekus H. (1972). On instabilities in Poiseuille and Couette flows of viscoelastic fluids. Prog. Heat Mass Transf..

[4] Groisman A., Steinberg V. (2000). Elastic turbulence in a polymer solution flow. Nature.

[5] Groisman A., Steinberg V. (2001). Stretching of polymers in a random three-dimensional flow. Phys. Rev. Lett..

[6] Liu Y., Steinberg V. (2010). Molecular sensor of elastic stress in a random flow. EPL.

[7] Groisman A., Steinberg V. (2004). Elastic turbulence in curvilinear flows of polymer solutions. New J. Phys..

[8] Latrache N., Crumeyrolle O., Abcha N., Mutabazi I. (2008). Destabilization of inertio-elastic mode via spatiotemporal intermittency in a Couette-Taylor viscoelastic flow. JPCS.

[9] Dutcher C.S., Muller S.J. (2013). Effects of moderate elasticity on the stability of co-and counter-rotating Taylor–Couette flows. J. Rheol..

[10] Arratia P.E., Thomas C.C., Diorio J., Gollub J.P. (2006). Elastic instabilities of polymer solutions in cross-channel flow. Phys. Rev. Lett..

[11] Haward S.J., Ober T.J., Oliveira M.S., Alves M.A., McKinley G.H. (2012). Extensional rheology and elastic instabilities of a wormlike micellar solution in a microfluidic cross-slot device. Soft Matter.

[12] Groisman A., Steinberg V. (2001). Efficient mixing at low Reynolds numbers using polymer additives. Nature.

[13] Burghelea T., Segre E., Bar-Joseph I., Groisman A., Steinberg V. (2004). Chaotic flow and efficient mixing in a microchannel with a polymer solution. Phys. Rev. E.

[14] Jun Y., Steinberg V. (2011). Elastic turbulence in a curvilinear channel flow. Phys. Rev. E.

[15] Tatsumi K., Takeda Y., Suga K., Nakabe K. (2011). Turbulence characteristics and mixing performances of viscoelastic fluid flow in a serpentine microchannel. JPCS.

[16] Rodd L.E., Scott T.P., Boger D.V., Cooper-White J.J., McKinley G.H. (2005). The inertio-elastic planar entry flow of low-viscosity elastic fluids in micro-fabricated geometries. J. Nonnewton. Fluid Mech..

[17] Rodd L.E., Cooper-White J.J., Boger D.V., McKinley G.H. (2007). Role of the elasticity number in the entry flow of dilute polymer solutions in micro-fabricated contraction geometries. J. Nonnewton. Fluid Mech..

[18] Gan H.Y., Lam Y.C., Nguyen N.T., Tam K.C., Yang C. (2007). Efficient mixing of viscoelastic fluids in a microchannel at low Reynolds number. Microfluid. Nanofluid..

[19] Lam Y.C., Gan H.Y., Nguyen N.T., Lie H. (2009). Micromixer based on viscoelastic flow instability at low Reynolds number. Biomicrofluidics.

[20] Rodd L.E., Lee D., Ahn K.H., Cooper-White J.J. (2010). The importance of downstream events in microfluidic viscoelastic entry flows: Consequences of increasing the constriction length. J. Nonnewton. Fluid Mech..

[21] Bonn D., Ingremeau F., Amarouchene Y., Kellay H. (2011). Large velocity fluctuations in small-Reynolds-number pipe flow of polymer solutions. Phys. Rev. E.

[22] Gan H.Y., Lam Y.C. (2012). Experimental observations of flow instabilities and rapid mixing of two dissimilar viscoelastic liquids. AIP Adv..

[23] Li F.C., Kinoshita H., Li X.B., Oishi M., Fujii T., Oshima M. (2010). Creation of very-low-Reynolds-number chaotic fluid motions in microchannels using viscoelastic surfactant solution. Exp. Therm. Fluid Sci..

[24] Grilli M., Vázquez-Quesada A., Ellero M. (2013). Transition to turbulence and mixing in a viscoelastic fluid flowing inside a channel with a periodic array of cylindrical obstacles. Phys. Rev. Lett..

[25] White J.L., Kondo A. (1977). Flow patterns in polyethylene and polystyrene melts during extrusion through a die entry region: Measurement and interpretation. J. Nonnewton. Fluid Mech..

[26] Nguyen H., Boger D.V. (1979). The kinematics and stability of die entry flows. J. Nonnewton. Fluid Mech..

[27] Lawler J.V., Muller S.J., Brown R.A., Armstrong R.C. (1986). Laser Doppler velocimetry measurements of velocity fields and transitions in viscoelastic fluids. J. Nonnewton. Fluid Mech..

[28] Yesilata B., Öztekin A., Neti S. (1999). Instabilities in viscoelastic flow through an axisymmetric sudden contraction. J. Nonnewton. Fluid Mech..

[29] Zhang H.N., Li F.C., Cao Y., Kunugi T., Yu B. (2013). Direct numerical simulation of elastic turbulence and its mixing-enhancement effect in a straight channel flow. Chin. Phys. B.

[30] Whalley R.D., Abed W.M., Dennis D.J.C., Poole R.J. (2015). Enhancing heat transfer at the micro-scale using elastic turbulence. TAML.

[31] Abed W.M., Whalley R.D., Dennis D.J., Poole R.J. (2016). Experimental investigation of the impact of elastic turbulence on heat transfer in a serpentine channel. J. Nonnewton. Fluid Mech..

[32] Ligrani P., Copeland D., Ren C., Su M., Suzuki M. (2018). Heat transfer enhancements from elastic turbulence using sucrose-based polymer solutions. J. Thermophys. Heat Transf..

[33] Taylor Z.J., Gurka R., Kopp G., Liberzon A. (2010). Long-duration time-resolved PIV to study unsteady aerodynamics. IEEE Trans. Instrum. Meas..

[34] Barnes H.A., Hutton J.F., Walters K. (1989). An Introduction to Rheology.

[35] Tamano S., Itoh M., Inoue T., Kato K., Yokota K. (2009). Turbulence statistics and structures of drag-reducing turbulent boundary layer in homogeneous aqueous surfactant solutions. Phys. Fluids.

[36] Japper-Jaafar A., Escudier M.P., Poole R.J. (2010). Laminar, transitional and turbulent annular flow of drag-reducing polymer solutions. J. Nonnewton. Fluid Mech..

[37] Kraichnan R.H. (1967). Inertial ranges in two-dimensional turbulence. Phys. Fluids.

[38] Leith C.E. (1968). Diffusion approximation for two-dimensional turbulence. Phys. Fluids.

[39] Batchelor G.K. (1969). Computation of the energy spectrum in homogeneous two-dimensional turbulence. Phys. Fluids.

[40] Raffel M., Willert C., Wereley S., Kompenhans J. (2007). Particle Image Velocimetry—A Practical Guide.

[41] Adrian R.J., Christensen K.T., Liu Z.C. (2000). Analysis and interpretation of instantaneous turbulent velocity fields. Exp. Fluids.

