# Supplementary material for: Elastic Turbulence of Aqueous Polymer Solution in Multi-Stream Micro-Channel Flow
Source: Micromachines (Basel). 2019 Feb 7;10(2):110. doi: 10.3390/mi10020110 (PMC6412290; doi:10.3390/mi10020110)
Supplement: Supplementary file 1 [file micromachines-10-00110-s001.zip › micromachines-421886-Supplementary Materials/micromachines-421886-Supplementary Materials.docx]

Supplementary Materials: Elastic Turbulence of Aqueous Polymer Solution in Multi-Stream Micro-Channel Flow

Jiayan Tai and Yee Cheong Lam

S1. Approximation of Liquid Properties in the Contraction

To approximate the amount of space occupied by the mainstream liquid in the contraction, numerical simulations in a straight channel (i.e. the contraction) were carried out using COMSOL Multiphysics, neglecting diffusion and elastic effects (see Figure S1). Similar to the experimental runs, the flow from each side-stream liquid was set to a quarter (i.e. 25%) of the total volume flow rate ($Q$). The straight channel had similar cross-sectional dimensions as the micro-channel contraction region, and upstream and downstream flows were neglected in the simulations. Fine meshing was employed, with element sizes ranging between 3 µm to 9 µm.

**Figure S1.** Numerical simulations of a 3-stream straight channel flow. The inset shows the cross-section of the 3-stream flow located 0.5 mm downstream from the junction. Red = center-stream and Blue = side-stream.

Firstly, the main-stream liquid was assumed to occupy 50% of the contraction space, and the average shear rate ($\dot{\gamma}$) was calculated (approximation based on the ratio of average velocity to half channel depth, i.e. $\dot{\gamma}$ = 2$Q$/(*d^2^w_c_*)). The corresponding viscosity ($\eta$) was then interpolated from the shear viscosity data (Figure 2 of manuscript) and input into the simulation. The 3-stream flow was modeled based on the Navier-Stokes equation (Equation (S1)) and the continuity equation (Equation (S2)):

| $\boldsymbol{\rho}\left[ \boldsymbol{\partial}\boldsymbol{U}\boldsymbol{/\partial}\boldsymbol{t}\mathbf{+}\boldsymbol{U\cdot}\boldsymbol{\nabla}\boldsymbol{U} \right]\boldsymbol{=-}\boldsymbol{\nabla}\boldsymbol{P+\eta}\boldsymbol{\nabla}^{\mathbf{2}}\boldsymbol{U}$ | (S1) |
| --- | --- |
| $\boldsymbol{\nabla}\boldsymbol{\cdot U=0}$ | (S2) |

For ease of computation, the convection-diffusion equation (Equation (S3)) was used to model viscosity as a species, which could be transported and diffused.

| $\boldsymbol{D}\boldsymbol{\nabla}^{\boldsymbol{2}}\boldsymbol{\eta=U\cdot}\boldsymbol{\nabla}\boldsymbol{\eta}$ | (S3) |
| --- | --- |

where D is the diffusion coefficient. By setting D to a small value (i.e. 10^−12^ m^2^/s), the test liquids were modeled as immiscible liquids. This was done to preserve the sharp interfaces between the 3 fluid streams (see Inset in Figure S1), for ease of computing the cross-sectional area occupied by each fluid. In addition, non-slip boundary conditions (Navier-Stokes equation) and insulation (Convection-Diffusion equation) were imposed on all walls.

From the simulation results, the average shear rate ($\dot{\gamma}$) could be extracted. This shear rate value was subsequently used to carry out a second interpolation on the shear viscosity plot (see Figure 2 of manuscript), to obtain the approximate shear-thinned viscosity ($\eta$) of the center-stream liquid in the contraction, which was input into the simulation again. After two simulation runs, the center-stream liquid was found to occupy ≈ 40% of the cross-sectional area ($A_{c}$) in the contraction. The average velocity of the center-stream liquid in the contraction ($\bar{U}$) was then computed i.e. $\bar{U}=0.5Q/A_{c}$.

S2. Supplementary Video Clip

To observe the 2D ‘sweeping’ motion of the center-stream liquid, particles were seeded into it only (i.e. no particles in side-stream liquid), and a video clip (recorded at 250 FPS) has been included to show the behavior of the center-stream liquid downstream of the contraction exit, at Q = 20 mL/h (see Sweeping_Motion.mp4 in Supplementary Materials). In the video clip, the seeded particles appear as white pixelated dots, while white borders correspond to the channel walls. As the center-stream liquid passes through the contraction, elastic energy is stored in the center-stream liquid, because the polymer molecules are highly stretched. Upon exiting the contraction, the highly elastic center-stream liquid releases its stored elastic energy, which manifests as a lateral 2D random ‘sweeping’ flow (can be observed in the video clip). This is possible since the center-stream liquid is not wall-bounded, but is sandwiched between two lower viscosity side-stream liquids (no particles) that act as soft ‘lubricating’ layers.

S3. Turbulent Intensities of Various Liquid Flows

Using the same micro-channel, we investigated the turbulent intensities for flows of various liquid combinations at Q = 20 mL/h i.e. 3-stream Newtonian (Re ≈ 7.2), 1-stream PEO (1 wt%, Re ≈ 0.8), and 1-stream DI Water (Re ≈ 41.4). For the 3-stream Newtonian flow, the center-stream and side-stream liquids used were 50 wt% and 25 wt% glycerol-water mixtures (Sigma Aldrich, Product number G7757) respectively. As observed in Figure S2, turbulent intensities for all other flow configurations are very much lower, in comparison to the 3-stream PEO flow configuration (obtained from Figure 8b of manuscript).


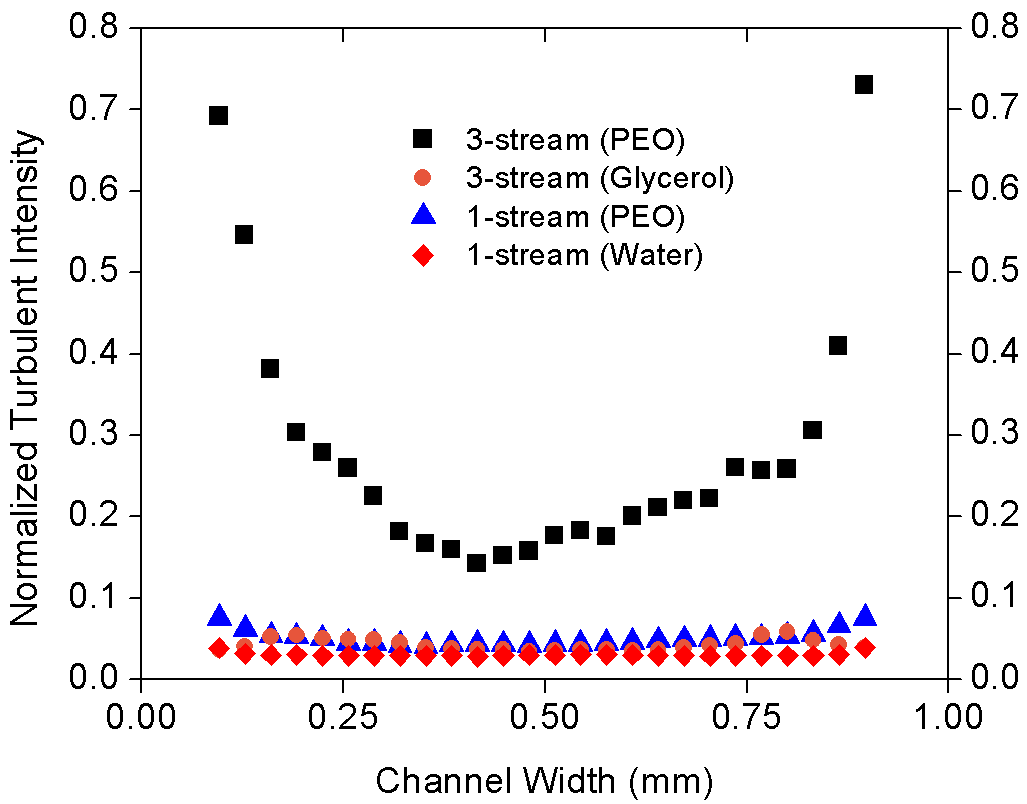


**Figure S2.** Turbulent intensities for various flow configurations. Data was obtained at *δ* = 1.0 mm. For comparison, the turbulent intensities of the 3-stream PEO flow (from Figure 8b of the manuscript) have also been superimposed.

S4. Validation of Taylor’s Hypothesis

To test the validity of the Taylor’s hypothesis, the 1-point temporal correlation function and the 2-point (spatial) correlation function were compared for the most turbulent flow state i.e. Q = 20 mL/h. The 1-point correlation function was computed based on the temporal velocity data (obtained using recording mode 2) at interrogation window A (*δ* = 0.3 mm, see Figure S3). In addition, the spatial correlation function was also evaluated from interrogation window A, with increasing spatial lags in the downstream direction (e.g. A-A’, see Figure S3). Each spatial lag corresponds to a resolution of 32 µm, which coincides with the interrogation window overlap spacing in the PIV analyses. Due to the limited field of view of the recording optics, the maximum correlation distance that could be evaluated was 1.0 mm downstream from interrogation window A.

**Figure S3.** Location of interrogation window A and direction of spatial lag. The blue arrow indicates the direction in which the spatial correlation function was evaluated.

Figure S4 shows the 1-point correlation function. The function decreases rapidly from unity to zero within a short period of time i.e. less than 0.05 s. Assuming that the Taylor’s hypothesis was valid, the temporal correlation function was used to obtain the approximated spatial correlation function (see Figure S5), via a simple operation involving the multiplication of time with the mean velocity.

**Figure S4.** Temporal autocorrelation function obtained using setup 2. Inset shows the magnified view from 0 to 0.2 s.

Figure S5 shows the calculated spatial correlation function, evaluated using the PIV measurements. The approximated spatial correlation function which was evaluated using the 1-point temporal correlation function, has also been superimposed onto Figure S5 for comparison. The results show that there is a reasonable agreement between the two correlation functions. Hence, the Taylor’s ‘frozen eddy’ hypothesis can be assumed to be valid in this study.


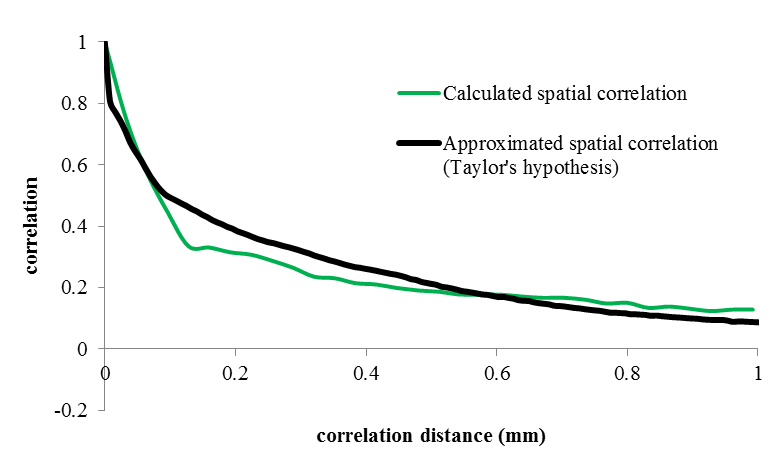


**Figure S5.** Comparison of spatial correlation function based on calculations and Taylor's hypothesis.
